# Impact of an educational workshop on psychiatrists’ attitude towards psychosomatic medicine

**DOI:** 10.1186/s12888-019-2424-9

**Published:** 2020-01-06

**Authors:** Franziska Baessler, Anja Ciprianidis, Fabienne Louise Wagner, Ali Zafar, Thanos Kanellopoulos, Tabea Chiara Baumann, Clara-Louisa Sandmann, Jobst-Hendrik Schultz

**Affiliations:** 1 Department of General Internal and Psychosomatic Medicine, Heidelberg University Hospital, Im Neuenheimer Feld 410, 69120 Heidelberg, Germany; 2 Mental Health Care Unit, Evgenidion Therapeftirion, National and Kapodistrian University of Athens, Athens, Greece

**Keywords:** Psychosomatics, Education, Trainees, Attitude, Innovative teaching

## Abstract

**Background:**

Although psychosomatic medicine is not recognised as a medical specialisation globally, it has proven useful for treating many disorders in Germany. This paper reports on the impact of an educational workshop as a tool for raising awareness about psychosomatic medicine among international psychiatrists.

**Methods:**

Psychiatrists from eight different countries were educated on psychosomatic medicine and psychotherapy during a 90-min workshop using a video, a slide presentation and an innovative teaching format called ‘speed coaching’. Learning outcomes were assessed by analysing questionnaires completed by the participants before and after the workshop.

**Results:**

Half of the participants who initially rejected the notion that psychosomatic medicine should be a specialisation on its own changed their minds in favour for it to be a separate discipline (*p* = 0.125). Awareness about which diseases and patients psychosomatic doctors deal with was increased. The intent for treatment of patients with eating disorders by a psychosomatic physician quadrupled among the participants (*p* = 0.004).

**Conclusions:**

A brief educational intervention can influence psychiatrists’ decisions to opt for approaches by doctors specialized in psychosomatics for certain disorders. Further studies may explore why psychiatrists agree or disagree that psychosomatic medicine should be a separate specialisation on its own.

## Background

‘Psychosomatics’, initially attributed to Johann Heinroth in 1818 [1], is recognised under different names such as biopsychosocial, integrative, holistic or patient-oriented medicine and most recently as consultation-liaison psychiatry [2–4]. As an interdisciplinary field, psychosomatic medicine provides an integrative view on health and disease, considering the interaction between biological, psychological and social variables [3, 5]. It refutes the dichotomy of psyche and soma to perceive and treat mind and body as a biological unity [6], reaffirming *who* the patient is as well as *what* disease they have [7]. This includes the study of psychosocial factors on the vulnerability and outcome of a medical disease and the integration of the psychological and psychiatric therapies into the treatment of the medical disease [5, 8].

At least since 2017, English-speaking countries have officially recognized the field as consultation-liaison psychiatry [2, 9–11] whereas continental Europe, especially Germany and France, continues to identify it as psychosomatic medicine [12]. In Germany, psychosomatic medicine has a large independent institutional base rooted in the traditional model of psychogenesis of disease and has developed well after integrating aspects of the biopsychosocial model [11, 13–15]. The psychogenetic model as the application of psychoanalysis in medicine was prevalent particularly in the US till the 1960s until critics, attributing its persisting popularity to tradition itself, termed it obsolete and incompatible with the psychosomatic doctrine of multi-causality [16–18]. Despite these reservations among the global academia, a PubMed search with the keyword ‘psychosomatic’ shows 3475 (11.78%) publications in German language, suggesting its continuing acceptance as an independent field. Currently only Germany and Japan recognise psychosomatic medicine as an independent specialisation outside psychiatry [13, 19].

As a consequence, the debate on psychosomatic medicine is replete with confusion and constantly misrepresented. This lack of global consensus has led to an underrepresentation of psychotherapy, social therapy and behavioural medicine [20]. Moreover, its importance in healthcare systems has been sparingly noted because of scepticism towards psychosomatic physicians and their reputation [21]. To initiate dialogue and develop consensus, it becomes essential to investigate what practicing physicians know about psychosomatic medicine and their attitude towards psychosomatic disorders. This study reports on the existing knowledge of international physicians, mainly practising psychiatrists, about psychosomatic medicine and investigates the effect of an educational intervention on their perception towards psychosomatic principles of healthcare.

## Methods

A brief workshop and pre- and post-surveys were conducted to educate psychiatrists from different countries on psychosomatic medicine. The pre- and post-intervention results were analysed to identify influential factors in forming opinions about psychosomatic medicine. Participation was voluntary and there were no exclusion criteria. All participants took the same intervention.

### Participants

All 160 physicians at the 2nd Congress of Early Career Psychiatrists titled ‘The Congress of All Young Psychiatrists Worldwide’ in Athens were invited to take part in the optional workshop. 18 participants voluntarily signed up for the workshop; they were not selected or recruited. The participants (male: 28.6%; female: 71.4%) were aged between 25 and 53 with a mean clinical experience of 3.45 years. Four participants were from Greece, three from Indonesia and one each from Cyprus, Latvia, New Zealand, Portugal, Russia and United Kingdom. Five participants did not state their nationality. Seven participants named ‘psychiatry’ as their speciality and one named ‘old age psychiatry’.

### Questionnaire

A semi-structured, anonymous questionnaire was used before and after the intervention to understand the attitudes and knowledge of psychosomatic medicine among the psychiatry community and to evaluate the workshop’s impact. The questionnaire included questions concerning the attitude on psychosomatic medicine, its knowledge and the workshop techniques. For the questionnaire, responses were given on a Likert scale [22], while for some questions the participants had to mark the correct choice or to fill in a descriptive field. All participants agreed to the anonymous scientific use of the data obtained.

### Workshop

A 90-min workshop titled ‘Highlights of Psychotherapy, Psychosomatics and Communication’ included different methods of knowledge transfer concerning psychosomatic medicine. It was coordinated and held by a psychiatrist trained in psychosomatic medicine with a master’s degree in medical education. During the intervention, a PowerPoint slide presentation, including text, pictures and videos, was used complemented by interactive workshop techniques. This consisted of group discussions in randomly chosen pairs of two, a large-group discussion and a therefore designed ‘speed coaching’ session similar to speed dating, wherein the candidates named their expertise in psychotherapy and then wrote the names of the participants they were interested in talking with.

The participants were randomly split in two groups with one consisting of those with expertise topic marked by others of interest and the other (named mentees) consisting of those without chosen expertise. All participants were matched by their expertise and focus of interest. They could arrange themselves on a first-come-first-serve basis. No more than two mentees were allowed per expert and the second mentee was only allowed after each expert had been matched with at least one mentee. In the end, each expert had one or two followers so the conversation between them continued in pairs of two or maximum three for a timeframe of around 5 min of ‘speed coaching’.

The presentation started with a five-minute documentary video titled ‘We treat the patient, not the disease’, which outlines the core of psychosomatic treatment at the oldest German psychosomatic clinic (available at https://www.klinikum.uni-heidelberg.de/Willkommen.1088.0.html). The film was followed by a case report from the Heidelberg University Hospital of a patient suffering from chronic pain for a couple of years experiencing psychological distress. The presentation continued afterwards with the explanation of the concept of simultaneous treatment (biological and psychological) of the Department for General Internal and Psychosomatic Medicine at the Heidelberg University Hospital. For example, for a patient suffering from tachycardia, an interdisciplinary, joint care by a cardiologist and a psychosomatic doctor was pointed out. The (psychotherapy) treatment of eating disorders was explained and the therapy for acceptance and commitment used for patients with chronic pain was shortly introduced.

### Data analysis

McNemar Test and Exact Wilcoxon Test were used to determine changes in attitudes of participants towards psychosomatic medicine and about which diseases should be treated by psychosomatic physicians (significance level α < 0.05). Answers of the open-ended questions were analysed descriptively. Microsoft Excel was used to analyse data.

## Results

The pre-and post-workshop opinions of participants to the question if ‘psychosomatic medicine should be a specialty of its own’ did not change significantly (*p* = 0.125). Pre-intervention 22.2% (*n* = 4) of the participants “agreed” or “strongly agreed” to psychosomatic medicine being a specialisation of its own. Post-intervention, the number of participants agreeing to the idea increased to 55.5% (*n* = 10). Table 1 summarises the results.

**Table 1:** Participants’ responses to the question if ‘psychosomatic medicine should be a specialty of its own’ before and after the workshop

	Agree	Disagree	Neutral	N/A	*p*-value
Pre-intervention	22.2% (*n* = 4)	22.2% (*n* = 4)	27.8% (*n* = 5)	27.8% (*n* = 5)	0.125
Post-intervention	55.5%. (*n* = 10)	16.7% (*n* = 3)	16.7% (*n* = 3)	11.1% (*n* = 2)	

For the disorders to be treated primarily by psychosomatic physicians, the perceptions changed significantly for eating disorders and somatoform disorders. Pre-intervention, 16.7% (*n* = 3) allocated eating disorders to a psychosomatic specialist whereas post-intervention, 61.1% (*n* = 11) shared this opinion. Pre-workshop, somatoform disorders were thought to be treated by psychosomatic doctors by 33.3% (*n* = 6) participants and post-workshop, it increased to 83.3% (*n* = 15). Table 2 provides an overview of the changes in their perception.

**Table 2:** Participants’ perception of which disorders should be treated by psychosomatic doctors before and after the workshop

Disorder	Treatment by psychosomatic doctor		
	Pre-workshop (%)	Post-workshop (%)	*p*-value
Chronic pain disorders	66.7	77.8	0.344
Depression	22.2	22.2	0.75
Eating disorders	16.7	61.1	0.004
Anxiety disorders	16.7	27.8	0.312
Somatoform disorders	33.3	83.3	0.002
Schizophrenia	0	0	n/a

Most participants stated their expertise to be in cognitive behavioural therapy. Their fields of interest included family therapy, existential psychotherapy and psychoanalysis. One-third of the participants were interested in psychodynamic psychotherapy. The trainings for becoming a psychotherapist varied between participants’ countries of origin. When asked about the training, one participant stated *“typically none … but there are master degrees in public and private universities and associations”*. Another participant answered *“six years of medicine to become a M.D. and four years of specialisation. Five years as psychotherapy student in university and specialising to get a certificate licence.”*

For an open-ended question about general ideas and thoughts about psychosomatic medicine, 11 participants gave an answer pre-intervention. After the workshop, 16 participants answered the question. All except one answered *“physical manifestation of psychological distress”*. The participant with a different answer associated it with disorders such as ‘oncological diseases and asthma’. Post-intervention answers were more complex and problems such as eating disorders or pain were named more often. One participant’s pre-intervention idea of psychosomatic medicine as *“the physical manifestation of psychological distress”* changed post-intervention to “*complex patients presenting with physical/medically unexplained symptoms that can be difficult to treat”*. Another participant responded *“…eating disorders (may be categorised under psychosomatic medicine)”*, which she earlier thought could be treated under psychiatry.

## Discussion

This is a first-of-its-kind survey to investigate the perceptions and knowledge of international physicians about psychosomatic medicine and whether or not an educational intervention can change their attitude towards the treatment of patients of certain disorders. The results suggest that a short intervention can positively affect attitudes and expectations towards psychosomatic medicine in line with research on creating awareness through brief interventions [23–25].

Although pre-and post-workshop opinions of participants about psychosomatic medicine as a separate specialisation did not change significantly statistically, half of the psychiatrists who initially rejected the idea changed their opinion in favour of it. This is important since psychosomatic medicine is now recognised as a psychiatry specialisation in a lot of countries and only a few countries provide an independent base for psychosomatic practice. Additionally, it suggests that significant gaps in knowledge about psychosomatic medicine may result in misconstrued opinions leading to criticism of the field [16].

Before psychosomatic medicine became consultation-liaison psychiatry, most psychiatrists were dissatisfied with its name which implied the field focused solely on somatoform disorders and was understood as such by doctors, trainees and patients [26]. In Germany, psychosomatic medicine has been recognised as a fundamental component of healthcare with an integrative approach towards organic and psychological problems [27]. It has a much larger independent institutional base compared to any other country [28, 29] partially due to resistance from psychiatry to integrate psychotherapy as a core method [15]. Its clinical core competency centres on integrated care for somatoform/functional disorders, eating disorders and somatopsychic disorders while overlapping with psychiatry for depression, anxiety and personality disorders [15].

In our study, the most significant changes in perception were observed for the participants agreeing that patients with eating and somatoform disorders needed psychosomatic intervention rather than psychiatric help. This hints at a more educated and differentiated view of the discipline post-intervention, as intended by the workshop. Understandably, the perceptions for treatment of chronic pain disorders and anxiety disorders did not change significantly, since the discussion surrounding these problems already overlaps with either internal medicine or psychiatry, respectively [17]. Similarly, the perceptions about treating depression and schizophrenia through psychosomatic medicine did not change at all. Even though treatment of depression is debated among psychosocial disciplines, it is generally understood as a disorder treated by doctors specialized in psychosomatics or psychiatry.

An interesting aspect of the qualitative analysis revealed that while many psychiatrists stated their expertise in cognitive behavioural therapy, about one-third of the participants were interested in psychodynamic psychotherapy. Recent developments considering psychosomatic medicine as a synonym for consultation-liaison psychiatry have run parallel to the declining trend of psychotherapy among psychiatrists, to which several factors have contributed [30–32]. There is enough evidence that psychotherapy treatment effects for most disorders are equivalent to or stronger than those of psychopharmacology [33]. A study reported about 81% psychiatrists in British Columbia continuing to integrate psychotherapy and pharmacotherapy in clinical practice [34]. While the proportion of time spent by psychiatrists practicing psychotherapy has diminished steadily over recent decades, the extent of this decline in non-English speaking countries is unclear.

Affecting the general attitude towards psychosomatic medicine (“psychosomatic medicine as a specialty of its own”) as well as efficacy expectations (“disorders to be primarily treated by psychosomatic doctors”) through workshops is a worthwhile endeavour, because it is a relatively simple and cost-effective way to address reservations and gaps in knowledge and education. Regarding the attitude of participants towards psychosomatic medicine as a specialty on its own, we cannot rule out a desirability bias towards “agree”. In addition, there is some missing data where participants chose not to answer this question. This could mean that these respondents had no strong opinion on this topic but there was a “neutral” answer choice as well. This suggests that there may have been changes in attitudes that we could not track through this assessment.

Although the results support previous studies, the generalizability of results could be termed limited because of the small sample size. However, it is ideal for such brief interactive educational workshops because too many participants result in a more theoretical teaching approach [35]. Further investigations should concentrate on why psychiatrists agree or disagree that psychosomatic medicine should be a separate specialisation. Assessing which instructional techniques are better for influencing opinions of the participants e.g. the film about a psychosomatic clinic, the enthusiasm of the instructor or the interaction with colleagues may also prove helpful. A follow-up study may help observe whether or not the intervention influenced the participants’ attitude in the long term and if it only changed their attitude but not their clinical behaviour.

## Conclusion

Considering the direction of healthcare provision owing to changes in lifestyle, psychosocial needs of chronic patients and shared-decision making, psychosomatic medicine still plays a significant therapeutic role beyond the reductionist biomedical and pharmacological approaches [17, 36]. The debate surrounding psychosomatic medicine has blurred the boundaries for several distinct psychosocial professions. Apparently psychiatrists are familiar with the model of biopsychosocial psychosomatic approach which resulted in the name change from psychosomatic to consultation-liaison psychiatry [2].

Our unique educational intervention proved successful in changing the perception and attitudes of international psychiatrists towards psychosomatic medicine and the treatment of patients of certain disorders by psychosomatic physicians. This might be helpful to increase the desirability for specialists, particularly in countries where psychosomatic medicine does not exist as a separate specialty. It would be wise to educate doctors about the fields of expertise in psychosomatic medicine and to address the need for psychodynamic approaches. Although the instructional methods constituting the workshop taken together were demonstrably effective, we have no evidence of the relative impact of these elements in isolation. For example, was the speed coaching technique useful in changing attitudes and increasing awareness or were the video portraits the actual active ingredient?

Further research could extend these findings through larger sample sizes and controlled instructional variables. The means of assessing knowledge may also be improved by more elaborate multi-item measures. This study adds to the techniques and interventions to target attitudes towards psychosomatic medicine in order to educate practitioners. These are important steps for the purpose of facilitating global recognition of psychosomatic medicine as an indispensable specialisation.

## Data Availability

Data can be made available by the corresponding author on reasonable request. No copyrights were used in the manuscript.

## Abbreviations

ABMS: American Board of Medical Specialties
US: United States
